# Anti-thrombotic strategies in patients with atrial fibrillation undergoing PCI

**DOI:** 10.1007/s00392-020-01708-8

**Published:** 2020-07-21

**Authors:** Andreas Schäfer, Ulrike Flierl, Johann Bauersachs

**Affiliations:** Department of Cardiology and Angiology, Hannover Medical School, Carl-Neuberg-Str. 1, 30625 Hannover, Deutschland

**Keywords:** Anti-platelet treatment, Anticoagulation, P2Y_12_ blocker, ASA, Triple therapy, Atrial fibrillation

## Abstract

Triple anti-thrombotic therapy combining oral anticoagulation and dual anti-platelet therapy following percutaneous coronary intervention in patients with atrial fibrillation was considered as standard and recommended by guidelines. While bleeding risk is considerable with that approach, data for efficacy are scare. Several trials assessed the possibility of reducing anti-thrombotic treatment by mainly shortening the exposure to acetylsalicylic acid. Dropping one of the anti-platelet components might increase the risk of stent thrombosis, myocardial infarction or stroke. Despite that fear, the recent trials’ primary endpoint was major and/or clinically-relevant non-major bleeding. We review data on major bleedings, intracranial bleedings and major adverse cardiovascular events from the published reports. We demonstrate that Non-Vitamin K oral anticoagulant (NOAC)-based strategies compared to VKA-based triple therapies significantly reduce the risk for TIMI-major bleedings by 39% and for intracranial bleedings by 66%, while they did not increase the risk for overall ischemic or embolic events. However, recent meta-analyses indicate an increased risk for stent thrombosis with less intense anti-thrombotic therapy. While the overall incidence rate for stent thrombosis is rather low, relative increases by about 30–60% are reported, but they did not translate into adverse clinical net-benefit ratios. This review highlights that using certain NOAC regimens proven effective for stroke prevention in AF can reduce the rate of bleeding without increasing ischemic or embolic events. Furthermore, additive ASA in triple anti-thrombotic regimens should be limited to 1 month and individual weighing of ischemic versus bleeding risk during the first 30 days seems to be reasonable.

**Electronic supplementary material:**

The online version of this article (10.1007/s00392-020-01708-8) contains supplementary material, which is available to authorized users.

## Introduction

Anti-thrombotic treatment in patients with atrial fibrillation (AF) undergoing percutaneous coronary intervention (PCI) harbours the risk of inducing relevant bleeding complications when an oral triple therapy is prescribed to prevent embolic stroke and coronary stent thrombosis at the same time [1]. Oral anticoagulation is highly efficient in reducing stroke risk in AF [2, 3], but inefficiently prevents stent thrombosis following PCI [4, 5]. On the contrary, dual anti-platelet therapy (DAPT) is highly efficient in preventing stent thrombosis following PCI [4, 5], but inefficiently reduces stroke risk in AF [6]. The dilemma is that for a long time, guidelines recommended triple anti-thrombotic treatment as the default strategy following PCI in AF to reduce the ischemic and embolic risks [7–9], but that strategy bears an enormous risk of relevant bleeding complications [10]. Over the last years, several studies have shown that more intense anti-thrombotic treatment in patients with acute coronary syndromes (ACS) does not automatically lead to fewer ischemic events [11–14] and less intense anti-thrombotic regimens were specifically tested after PCI in AF patients [15–18]. As most of the studies are not comparable between each other, we want to highlight the individual specificities of the trials and to compare similar outcome data where available.

## Preventing stent thrombosis following percutaneous coronary intervention

Anti-platelet therapy is a corner stone in acute and chronic treatment of ischaemic cardiovascular diseases comprising stable coronary artery disease (CAD) as well as ACS. DAPT is required for a defined period of time following stent implantation during elective (usually 6 months) as well as urgent (usually 12 months) PCI to prevent stent thrombosis and subsequent myocardial infarction [19]. Generally, clopidogrel is the most commonly used P2Y_12_ inhibitor prescribed together with acetylsalicylic acid (ASA) for DAPT following elective PCI. The European Society of Cardiology (ESC) published a “Focused Update DAPT 2017”, which summarises the current standard in individualised anti-platelet therapy [19]. Current ESC guidelines (2018 myocardial revascularisation [8], 2015 NSTE-ACS [20], 2017 STEMI [21], 2017 DAPT [19]) demand an increasingly differentiated anti-thrombotic treatment for ACS patients to maximise treatment efficacy and to reduce bleeding risk. Nevertheless, triple therapy is still the default option following PCI in patients on oral anticoagulation (OAC), at least in European recommendations [8].

When bleeding is a serious concern, shortening DAPT duration even to only 1 month while using modern drug-eluting stents, did not increase the rate of stent thrombosis or myocardial infarction in the ZEUS trial compared to short DAPT duration with the use of bare-metal stents [22]. Therefore, operators are encouraged to use drug-eluting stents also in patients with uncertain ability for longer DAPT treatment. Current guidelines recommend to shorten DAPT duration, if clinical co-variables indicate a potential bleeding risk, e.g. if the PRECISE-DAPT score is ≥ 25 [8, 19].

In the early times of PCI, several trials documented that OAC using vitamin K antagonists (VKA) even with target INRs above 2.0–3.0 were not as efficient as DAPT consisting of ASA and ticlopidine in preventing stent thrombosis but caused much higher rates of severe bleeding [4, 5]. Therefore, when a patient has an indication for OAC, OAC alone might not be sufficient to prevent stent thrombosis after PCI. However, it is often forgotten that those older trials did not use VKA alone, but combined VKA with ASA indicating that ASA might not have such a great effect on the prevention of stent thrombosis as it was used in both groups, those with and those without stent thrombosis occurring [4, 5].

## Preventing strokes and systemic embolism in atrial fibrillation

Ischemic or embolic strokes are the major disabling complications in patients with atrial fibrillation (AF). OAC can prevent most of these events and is, therefore, widely used for stroke prevention in AF (SPAF) [23]. Excluding patients with very low stroke risks, the superiority of OAC compared to non-treatment is overwhelming, and based on current ESC “2016 Guidelines for the management of atrial fibrillation”, should be used in SPAF [9]. Furthermore, the European Heart Rhythm Association (EHRA) published their “2018 EHRA Practical Guide on the use of non-vitamin K antagonist oral anticoagulants in patients with atrial fibrillation”, which elucidates the advantage of non-vitamin K oral anticoagulants (NOACs) compared to vitamin K antagonists (VKA) [24]. Previously, a potential benefit of certain NOACs compared to VKA has been described in a meta-analysis including differing indications for anticoagulation [25]. NOACs are safe and effective during cardioversion [26–28], AF ablation [29–32], following transcatheter aortic valve implantation [33] and even when left atrial appendage thrombi have been formed on OAC their resolution appears to be not different on NOACs and VKA [28, 34, 35]. Upon availability of NOACs in Germany, AF patients were more frequently anticoagulated and stroke rate decreased without an increase in bleeding complications [36] and may exert differential effects on cardiac function than VKA [37].

ASA had previously been considered as a potential alternative to OAC in elderly patients due to a presumed lower bleeding risk than VKA. In the AVERROES trial randomising 5,599 patients to apixaban vs ASA, apixaban had a similar bleeding risk compared to ASA, but reduced stroke and systemic embolism (SSE) by 55% [38]. More specifically, in elderly patients apixaban had a similar bleeding risk and reduced embolic risk compared to ASA dramatically (e.g. 86% risk reduction for patients ≥ 85 years of age) [39]. Consequently, current guidelines clearly state that single anti-platelet treatment is not recommended for SPAF [9]. DAPT is not an option to replace OAC for SPAF in general due to its limited efficacy resulting in a 44% higher ischemic event rate than VKA in the ACTIVE W trial randomising 6706 patients either to DAPT combining acetylsalicylic acid (ASA) plus clopidogrel or VKA [6]. DAPT with more potent P2Y_12_ inhibitors has not been evaluated for SPAF.

## Incremental harm of conventional triple anti-thrombotic therapy

When a patient with AF requires PCI with consecutive DAPT, combining ASA + OAC + clopidogrel can increase the risk of bleeding by 3–4-fold compared to OAC alone [10, 40]. Thus, there had been a quest for alternative anti-thrombotic combination therapies with less bleeding risk. First, gastrointestinal bleeding is reduced by proton-pump inhibitors whenever a combination of anti-platelet and OAC medications is prescribed [41, 42]. Second, a combination of OAC with either prasugrel or ticagrelor is not recommended due to an increased bleeding risk [19, 43, 44]. Third, choosing a NOAC instead of VKA might reduce the bleeding risk as all four NOAC strategies tested in and approved for SPAF use an anti-thrombotic intensity that is comparable to sub-therapeutic dosing of low-molecular weight heparins or VKA. Fourth, dropping one out of three anti-thrombotic drugs early after PCI might further reduce bleeding risk, if it is safe regarding prevention of stent thrombosis. As trials mentioned in the upcoming sections have different bleeding definitions, the different definitions are summarized in Table 1.

**Table 1:** Definition of bleeding criteria as used in cited trials

	BARC [64] Bleeding Academic Research Consortium	GUSTO [65] Global Use of Strategies To Open coronary arteries	ISTH [54, 66] International Society on Thrombosis and Haemostasis	PLATO [67] Platelet Inhibition and Patient Outcomes	TIMI [53] *Thrombolysis In Myocardial Infarction*
Definition of major bleeding	*BARC 5* • fatal bleeding • definite: overt bleeding, autopsy, imaging • probable: clinically suspicious for bleeding without autopsy or imaging *BARC 4* • CABG-related bleeding • Intracranial ≤ 48 h • Reoperation to control bleeding • Transfusion ≥ 5 units of whole blood or red cells ≤ 48 h • Chest tube output ≥ 2 L ≤ 24 h *BARC 3:* • Overt bleeding + haemoglobin drop ≥ 3 g/dL • Any transfusion with overt bleeding • Cardiac tamponade • Requiring surgical control • Requiring intravenous vasoactive agents • Intracranial • Intraocular bleed comprising vision	*Severe or life-threatening bleeding* • Intracerebral • Resulting in substantial haemodynamic compromise requiring treatment *Moderate bleeding* • Requiring transfusion	• Fatal bleeding • Symptomatic bleeding in a critical area or organ such as intracranial, intraspinal, intraocular, retro-peritoneal, intraarticular or pericardial, or intramuscular with compartment syndrome • Bleeding causing a fall in haemoglobin level ≥ 2 g/dL, or leading to transfusion ≥ 2 units of whole blood or red cells	*Major life-threatening bleeding* • Fatal bleeding • Intracranial bleeding • Intrapericardial bleeding with cardiac tamponade • Hypovolemic shock or severe hypotension due to bleeding requiring pressors or surgery • Decline in haemoglobin level ≥ 5.0 g/dL • Transfusion of at least 4 units of red cells *Other major bleeding* • Bleeding leading to clinically significant disability (e.g., intraocular bleeding with permanent vision loss) • Bleeding either associated with a drop in the haemoglobin level ≥ 3 g/dL (< 5 g/dL) • Requiring transfusion of 2 to 3 units of red cells	• Reduction of haemoglobin ≥ 5 g/dL (or > 15% in haematocrit) • Any intracranial bleeding
Definition of non-major bleeding	*BARC 2* Any overt, actionable sign of haemorrhage (e.g. more bleeding than would be expected for a clinical circumstance, including bleeding found by imaging alone) that does not fit the criteria for BARC 3—5 but does meet at least one of the following criteria: • Requiring nonsurgical, medical intervention by a healthcare professional • Leading to hospitalization or increased level of care • Prompting evaluation *BARC 1* Bleeding that is not actionable and does not cause the patient to seek unscheduled performance of studies, hospitalisation, or treatment by a healthcare professional; may include episodes leading to self-discontinuation of medical therapy by the patient without consulting a healthcare professional	*Minor bleeding* Other bleeding not requiring transfusion or causing haemodynamic compromise	*Clinically-relevant non-major bleeding* Any sign or symptom of haemorrhage (e.g., more bleeding than would be expected for a clinical circumstance, including bleeding found by imaging alone) that does not fit the criteria for the ISTH definition of major bleeding but does meet at least one of the following criteria • Requiring medical intervention by a healthcare professional • Leading to hospitalization or increased level of care • Prompting a face to face (i.e., not just a telephone or electronic communication) evaluation	*Minor bleeding* Any bleeding requiring medical intervention but not meeting the criteria for major bleeding	• Observed blood loss and a drop in haemoglobin of 3 to 5 g/dL (or 10% to 15% in haematocrit) from study entry to the time of the lowest haemoglobin within 10 days • Spontaneous gross haematuria or hematemesis (> 120 ml), even if the haemoglobin or haematocrit drop was less than 3 g/dL or less than 10%, respectively • Unobserved loss ≥ 4 g/dl in haemoglobin or ≥ 12% in haematocrit Blood loss attributable to revascularization or other surgical procedures was not classified as a TIMI haemorrhagic event

There have been several trials comparing additive NOACs in addition to DAPT in patients with PCI for ACS, and the results are diverse. In RE-DEEM, adding dabigatran to DAPT was associated with an increased ischaemic event rate despite more intense anti-thrombotic therapy on lower doses and more ISTH-major bleedings on the usual dabigatran doses [14]. The mechanism of increased rates of myocardial infarctions on dabigatran as described in the RE-LY trial for SPAF [45] or in meta-analyses including other direct thrombin inhibitors [46] is still under debate, however, functional experiments suggest that there is a distinct mechanism in direct thrombin inhibitors which contributes to increased platelet activation and adhesion [47]. In ATLAS-ACS 2 TIMI 51 adding rivaroxaban 2 × 2.5 mg/day or 2 × 5 mg/day to DAPT, ischaemic events were lower on additive rivaroxaban, but non-CABG-associated TIMI-major bleedings were increased to a similar extent as ischaemic events were lowered. It is important to note that neither of the two bi-daily dosing regimens had ever been proven effective for SPAF [48]. The APPRAISE-2 trial adding full-dose apixaban to DAPT was terminated prematurely because of an increase in major bleeding events with apixaban in the absence of a counterbalancing reduction in recurrent ischemic events [12]. In summary, there is no evidence of clinical net-benefit by adding OAC with NOACs on top of DAPT in ACS patients. In most studies, there is even an increase in harm measured by major bleeding complications.

## De-escalating platelet inhibition in patients anticoagulated for SPAF

**Fig. 1:**
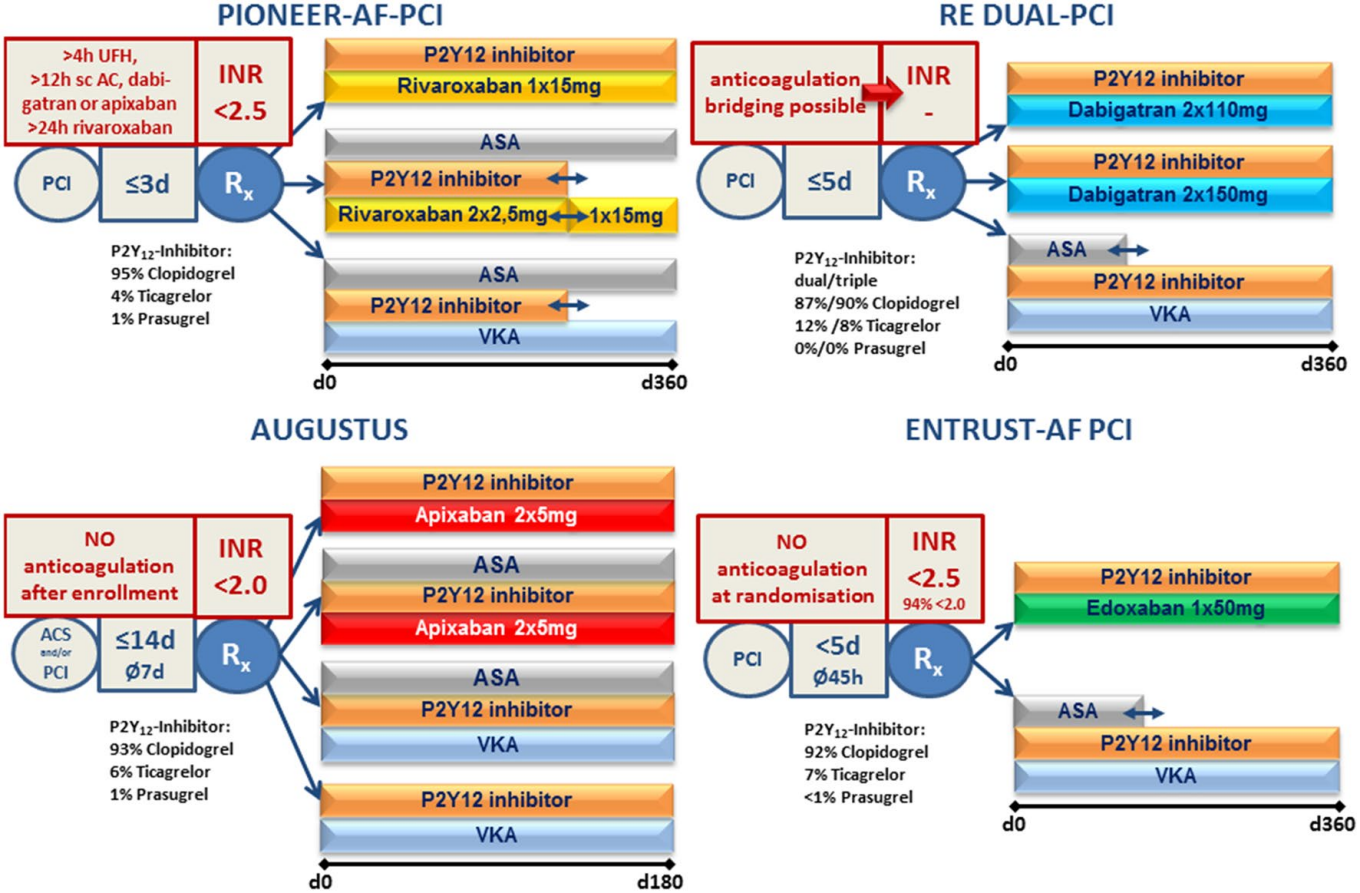
Design of the four NOAC trials using less intense anti-thrombotic strategies following PCI and/or ACS in patients with indication for anticoagulation for stroke prevention in atrial fibrillation. *ACS* acute coronary syndrome, *ASA* acetylsalicylic acid, *INR* international normalised ratio, *NOAC* non-vitamin K oral anticoagulant, *PCI* percutaneous coronary intervention, *sc AC* subcutaneous anticoagulation, *VKA* vitamin K antagonist. In PIONEER AF-PCI and RE DUAL-PCI the average duration from PCI to randomisation has not been reported

Five studies have investigated strategies of early discontinuation or even only peri-procedural application of ASA in patients receiving a P2Y_12_ inhibitor with OAC: WOEST [49] dropped ASA on a VKA background, whereas PIONEER AF [16], RE-DUAL PCI [15], AUGUSTUS [17], and ENTRUST-AF PCI [18] evaluated dual strategies on the different NOAC backgrounds (Fig. 1, Table 2) [50, 51]. However, all studies were not sufficiently powered to demonstrate efficacy regarding stroke prevention. Using a network meta-analysis of these trials, there was no indicator of increased ischaemic risk when using a NOAC in combination with only a P2Y_12_ inhibitor. In particular, there was no increase in stroke risk when using a NOAC-based dual anti-thrombotic regimen compared to a VKA-based triple regimen [18, 52]. The specific trials are discussed in more detail:

**Table 2:** Characteristics of trials combining anticoagulants and platelet inhibitors after percutaneous coronary interventions

	Triple anti-thrombotic treatment	Dual anti-thrombotic treatment	Additional arm	Patients (*n*)	Mean age (years)	Male sex, *n* (%)	Previous stroke, *n* (%)	CHA_2_DS_2_-VASc	HAS-BLED
Pioneer AF [16]	VKA INR 2–3 + P2Y_12_ inhibitor + acetylsalicylic acid	Rivaroxaban 1 × 15 mg + P2Y_12_ inhibitor	Rivaroxaban 2 × 2.5 mg + acetylsalicylic acid + P2Y_12_ inhibitor	2124	70	1582 (74%)	0 (0%)	3.7 ± 1.6	3.0 ± 0.9
Re-dual PCI [15]	VKA INR 2–3 + P2Y_12_ inhibitor + acetylsalicylic acid	Dabigatran 2 × 150 mg + P2Y_12_ inhibitor	Dabigatran 2 × 110 mg × + P2Y_12_ inhibitor	2725	69/72*	2070 (76%)	226 (8.3%)	3.7 ± 1.5	2.7 ± 0.7
Augustus [17]	VKA INR 2–3 + P2Y_12_ inhibitor	Apixaban 2 × 5 mg + P2Y_12_ inhibitor	Second randomisation ± acetylsalicylic acid	4614	71	3277 (71%)	633 (13.8%)	3.9 ± 1.6	2.9 ± 0.9
Entrust-AF [18]	VKA INR 2–3 + P2Y_12_ inhibitor + acetylsalicylic acid	Edoxaban 1 × 60 mg + P2Y_12_ inhibitor	–	1506	70	1120 (74%)	189 (12.5%)	4.0 (3·0–5·0)	3.0 (2·0–3·0)

### VKA in WOEST

The WOEST trial, an open-label, randomised, controlled trial in 15 centres in Belgium and the Netherlands, investigated 573 patients (mean age 70 years; 36% PPI) requiring OAC (11% mechanical valve prosthesis; 20% apical aneurysm, pulmonary embolism, peripheral arterial disease or left-ventricular ejection fraction < 30%; 69% AF; mean CHADS_2_-score: 1.6). Following PCI, they were treated either with triple therapy consisting of VKA, clopidogrel and ASA or with the combination of OAC with clopidogrel; patients not pre-treated with ASA received a loading-dose of 320 mg ASA at the time of PCI. Randomisation occurred prior to or within 4 hours following PCI. The primary safety endpoint was any bleeding within one year. Any bleeding occurred in 44.4% of patients on triple therapy, but only in 19.4% on dual therapy (risk reduction 64%, *p* < 0.0001). The rate of severe and moderate bleedings according to GUSTO-criteria was significantly reduced from 12.3% to 5.4% (risk reduction 58%, *p* = 0.003). The rate of major- and minor-bleedings according to TIMI criteria was significantly reduced from 31.3% to 14.0% (risk reduction 60%, *p* < 0.0001). Due to the rather low number of study participants, the effect on severe bleedings was statistically not significant, but showed a similar numeric trend: major bleeding by TIMI criteria were lowered from 5.6 to 3.2% (reduction by 44%, p =0.159), severe bleedings according to GUSTO-criteria were lowered from 3.5% to 1.4% (reduction by 60%, p=0.119). The rate of blood transfusion was significantly reduced from 9.5% to 3.9% (reduction by 61%, *p* = 0.011). Dropping ASA did not increase ischemic events, in contrast the dual combination of OAC + clopidogrel significantly reduced the secondary endpoint consisting of death, myocardial infarction, stroke, target-lesion revascularisation and stent thrombosis from 17.6 to 11.1% (reduction by 40%, p=0.025) compared to conventional triple therapy. Mortality was significantly reduced (6.3% vs. 2.5%, reduction by 61%, *p* = 0.027) on dual therapy and stent thrombosis was numerically lower (3.2% on triple vs. 1.4% on dual therapy, reduction 56%, p=0.165) [49].

In summary, WOEST encouraged that dropping ASA very soon after peri-procedural application might lower bleeding rates and is most probably not associated with increase in ischemic events. In contrast to the subsequent NOAC trials, however, OAC was uninterrupted as patients had an indication for OAC, were on VKA prior to PCI and were all continued on VKA thereafter. While this trial was the first to investigate dual compared to triple anti-thrombotic treatments, it was also by far the smallest. Therefore, conclusions on ischemic event rates have to be considered with caution.

### Rivaroxaban in PIONEER AF-PCI

The PIONEER AF-PCI trial, an open-label, randomised, multicentre trial compared triple therapy consisting of VKA, P2Y_12_ inhibitor (mainly clopidogrel) and ASA to the factor Xa-antagonist rivaroxaban (with a lower dose than approved for SPAF of 1 × 15mg/day instead of 1 × 20mg/day for duration of combination therapy, or 1 × 10mg/day if creatinine clearance was impaired at 30–50 ml/min) in combination with a P2Y_12_ inhibitor or rivaroxaban in a low dose of 2 × 2.5mg/day in combination with ASA and a P2Y_12_ inhibitor in 2124 patients. All patients not pre-treated with ASA received at least one dose at the time of PCI. The primary safety endpoint was clinically significant bleeding within one year (a composite of major bleeding or minor bleeding according to TIMI criteria [53] or bleeding requiring medical attention). This occurred in 26.7% of patients on triple therapy, in 16.8% (risk reduction vs VKA 41%, *p* < 0.0001) on dual therapy with rivaroxaban 1x15mg/day plus P2Y_12_ inhibitor, and in 18.0% (risk reduction vs VKA 37%, p<0.0001) on triple therapy with rivaroxaban 2 × 2.5mg/day plus ASA plus P2Y_12_ inhibitor. Due to the low number of study participants, the effect on major bleedings was statistically not significant, but showed a similar numeric trend. Ischemic and embolic events (death, MI, stroke) occurred to a similar extent, but due to the low number of study participants and low ischemic event rates, a statistically valid analysis cannot be made. Nevertheless, there are two major safety concerns regarding the PIONEER AF-PCI trial: first, as the investigated doses of rivaroxaban had not been proven to be effective for SPAF before, highest risk patients with a history of stroke or TIA were excluded from the trial; second, in patients with moderate-high risk for stroke (CHADS_2_-score ≥ 2), ischemic strokes were increased from 0.3% on VKA-based triple therapy to 1.5% on the dual strategy using rivaroxaban 1 × 15mg/day plus clopidogrel and 1.4% on the triple strategy using rivaroxaban 2 × 2.5mg/day plus DAPT [16].

In summary, PIONEER AF-PCI did evaluate dose regimes of rivaroxaban, which had not been proven effective for SPAF before. Patients at moderate-high risk showed a trend towards more ischemic strokes. Therefore, at least in AF patients at moderate-to-high risk for stroke the investigated strategies cannot be considered as an adequate alternative to VKA-based triple therapy due to lack of proven equivalent in stroke prevention.

### Dabigatran in RE-DUAL PCI

The RE-DUAL PCI trial, an open-label, randomised, multicentre trial compared triple therapy consisting of VKA, ASA (1 month after bare-metal-stent, 3 months after drug-eluting stent) and a P2Y_12_ inhibitor (mainly clopidogrel) to the thrombin antagonist dabigatran (2 × 150mg/day or 2 × 110mg/day randomised to VKA in a 1:1:1 fashion) in combination with a P2Y_12_ inhibitor in 2725 patients. All patients not pre-treated with ASA received at least one dose at the time of PCI. All patients in the US as well as non-elderly patients (< 70 years in Japan, < 80 years outside Japan and US) were randomised 1:1:1 to the groups mentioned above, elderly patients outside the US were randomised 1:1 to either VKA-based triple therapy or dabigatran 2 × 110mg/day. Based on these differing age criteria, there are two different VKA-triple therapy groups in comparison to either dose of dabigatran. The primary safety endpoint was clinically significant bleeding within one year according to ISTH criteria [54]. It was lower on dual therapy with dabigatran 2 × 110mg/day plus P2Y_12_ inhibitor occurring in 15.4% compared to VKA-based triple therapy in 26.9% of patients, (risk reduction by 48%, *p* < 0.0001). On dual therapy with dabigatran 2 × 150mg/day plus P2Y_12_ inhibitor, the primary safety endpoint occurred in 20.2% compared to 25.7% on VKA-based triple therapy, (risk reduction by 28%, *p* = 0.002). Using dabigatran 2 × 110mg/day in dual therapy lowered the primary endpoint from 26.9% on VKA-based triple therapy to 15.4% (risk reduction by 48%, *p* < 0.001). The rate of major bleedings according to ISTH- and TIMI criteria was significantly reduced by both dual anti-thrombotic dabigatran regimes. Ischemic or embolic events occurred more often on dabigatran 2 × 110mg/day compared to VKA-based triple therapy. Patients on dabigatran 2 × 110mg/day had numerically higher rates of death (5.6% vs. 4.9%), myocardial infarction (4.5% vs. 3.0%), stroke (1.7% vs. 1.3%) and stent thrombosis (1.5% vs. 0.8%); more specifically, the predefined criterion for non-inferiority regarding a composite ischemic endpoint was not met on dabigatran 2 × 110mg [15].

In summary, RE-DUAL PCI showed a potential of lower bleeding with similar ischemic risk when using the higher of the two approved doses of dabigatran. The lower dose indicated a potentially increased ischemic risk. As elderly patients were treated with the lower dose, potentially there might be a useful indication for younger patients treated with the higher dose of dabigatran, but dabigatran should not be the default anti-thrombotic combination strategy in elderly patients.

### Apixaban in AUGUSTUS

The AUGUSTUS trial, an open-label, randomised, multicentre trial first randomised patients to either OAC with VKA or the factor Xa-antagonist apixaban (in the dose approved for SPAF of 2 × 5mg/day or dose reduction to 2 × 2.5mg/day, if the usual dose reduction criteria applied [55]) in 4614 patients. In a second step, patients were then additionally randomized to receive ASA 100 mg/day or placebo on maintenance therapy. All patients not pre-treated with ASA received at least one dose at the time of PCI prior to randomisation. Elective PCI was performed in 38.8%, PCI in ACS in 37.3%, and conservatively treated ACS occurred in 23.9% of study participants. The primary safety endpoint was clinically significant bleeding within 6 months (a composite of major bleeding or clinically-relevant non-major bleeding according to ISTH criteria [54]). This occurred in 10.5% on apixaban compared to 14.7% on VKA (risk reduction 31%, *p* < 0.001). The rate of ISTH- and TIMI-major bleedings was lower on apixaban than on VKA. Ischemic and embolic events occurred in similar rates on both anticoagulant strategies. Regarding the randomisation to ASA vs. placebo, the primary safety endpoint occurred in 16.1% on ASA compared to 9.0% on placebo (risk increase 89%, *p* < 0.001). The rate of ISTH- and TIMI-major bleedings was increased by additive treatment with ASA, while ischemic and embolic events were not significantly reduced [17].

When analysing the combined randomisations, the lowest rate for major or clinically-relevant, non-major bleeding according to ISTH criteria was observed on apixaban without ASA (7.3%), followed by VKA without ASA (10.9%), Apixaban with ASA (13.8%) and VKA with ASA (18.7%). The lowest rate of major bleeding according to ISTH criteria was observed on apixaban without ASA (2.0%), followed by VKA without ASA (3.9%), apixaban with ASA (4.2%) and VKA with ASA (5.5%). The lowest rate of death and ischemic events was observed on VKA with ASA (5.7%), followed by apixaban with or without ASA (each 6.2%) and VKA without ASA (7.3%). Most interestingly, there appeared to be no difference in ischemic/embolic events with or without ASA, if apixaban was used as the anticoagulant in contrast to VKA [17]. Of note, a P2Y_12_ inhibitor was used in all groups.

Within the first 30 days in AUGUSTUS there was a balancing of more severe bleedings [fatal, intracranial, and other ISTH-major; absolute risk difference +0.97 (95% CI 0.23–1.70)] compared to fewer severe ischemic events [CV death, stent thrombosis, myocardial infraction, stroke (absolute risk difference − 0.91 (95% CI − 1.74 to − 0.08)] on additive ASA. Beyond 30 days, additional ASA did not reduce severe ischemic events [− 0.17 (95% CI − 1.33 to 0.98)], but increased severe bleedings [+1.25 (95% CI 0.23–2.27)] [56].

In summary, AUGUSTUS demonstrated good safety for the approved SPAF dose of apixaban in comparison to VKA. Overall ischemic events were not meaningfully higher on apixaban + P2Y_12_ inhibitor as on apixaban-based triple therapy. However, the pure PCI data have not been shown yet. The window from qualifying event to randomisation is the longest in all studies.

### Edoxaban in ENTRUST-AF PCI

The ENTRUST-AF PCI trial, an open-label, randomised, multicentre trial compared triple therapy consisting of VKA, P2Y_12_ inhibitor (mainly clopidogrel) and ASA (for 1–12 months guided by clinical presentation with either stable coronary artery disease or ACS) to the factor Xa-antagonist edoxaban (in the dose approved for SPAF of 1 × 60mg/day or with dose reduction to 1 × 30mg/day, if creatinine clearance ≤ 50 ml/min, body weight ≤ 60 kg, or concomitant therapy with certain *P*-glycoprotein inhibitors [57]) in combination with a P2Y_12_ inhibitor in 1506 patients. All patients not pre-treated with ASA received at least one dose at the time of PCI. The primary safety endpoint was clinically significant bleeding within 1 year (a composite of major bleeding or clinically-relevant non-major bleeding according to ISTH criteria [54]).

This occurred in 25.6%/years on triple therapy and in 20.7%/year on dual therapy with edoxaban 1x60mg/day plus P2Y_12_ inhibitor (risk reduction vs VKA 17%, *p* < 0.001 for non-inferiority, *p* = 0.1154 for superiority). ISTH-major bleedings occurred in 7.2%/year on VKA-based triple therapy and in 6.7%/year on edoxaban-based dual therapy (reduction by 5%, *p *= 0.7865). Ischemic and embolic events (death, MI, stroke, systemic embolism, definite stent thrombosis) occurred in 6.9%/year on triple therapy using VKA and in 7.3%/year on dual therapy with edoxaban (hazard ratio 1.06 (0.71–1.69), no *p* value reported).

The ENTRUST-AF PCI trial had about the shortest time from PCI to randomisation of the modern trials, most patients were randomised in less than 48 h. 94% had an INR < 2.0 at randomisation, even after one week, 42% of VKA-treated patients were below an INR of 2.0. Therefore, during the initial days the edoxaban dual-therapy group actually had still some ASA effect, whereas the labelled VKA-triple therapy group was a DAPT group in about half the patients. This might have influenced the primary endpoint of major or clinically-relevant, non-major bleeding, which was higher in the edoxaban group early during the trial and substantially lower than VKA-triple therapy after day 14.

In summary, on the long observation period of 1 year dual treatment with a factor Xa-antagonist in a SPAF-approved dose and P2Y_12_ inhibitor caused less bleeding as VKA-based triple therapy. These findings are consistent between AUGUSTUS and ENTRUST-AF PCI. Whether ASA should be given for some days early on was intensely debated after the numerically higher incidence of ischemic events during the first week in ENTRUST-AF PCI.

## Dual strategies in elderly patients

In elderly patients, NOACs may be related to less side effects due to medication interaction than VKA. For most of the above mentioned trials, the primary bleeding endpoint has been reported for elderly patients, but for major bleedings or ischemic events there is yet insufficient data reported [58]. In general, clinically significant bleedings in elderly patients were lower on NOAC/dual compared to VKA/triple strategies [15–18, 49]. In summary, it seems reasonable for elderly patients with AF, who require PCI, to administer ASA peri-procedurally followed by OAC plus clopidogrel. OAC can principally mean NOAC, but limitations apply to the currently published studies with dabigatran (trend towards more ischemic events in lower dose, only lower dose for elderly patients) and rivaroxaban (excluding patients with higher stroke risk, dose reduction without proven clinical efficacy) [59]. Data for apixaban do suggest that full-dose NOAC instead of VKA is safe and efficient in elderly patients. Current ESC recommendations emphasize that if NOACs are used they should be given without empiric dose reduction [19]. The advantage of all combinations of NOAC and clopidogrel are significantly lower bleeding rates compared to conventional triple therapy using VKA [59]. Another important contribution to prophylaxis of bleeding especially for elderly patients is to avoid permanent combinations of ASA or clopidogrel with (N)OAC for CAD and AF following the usual post PCI duration of DAPT or additive P2Y_12_ inhibition [9, 19].

## Risk–benefit evaluation

**Fig. 2:**
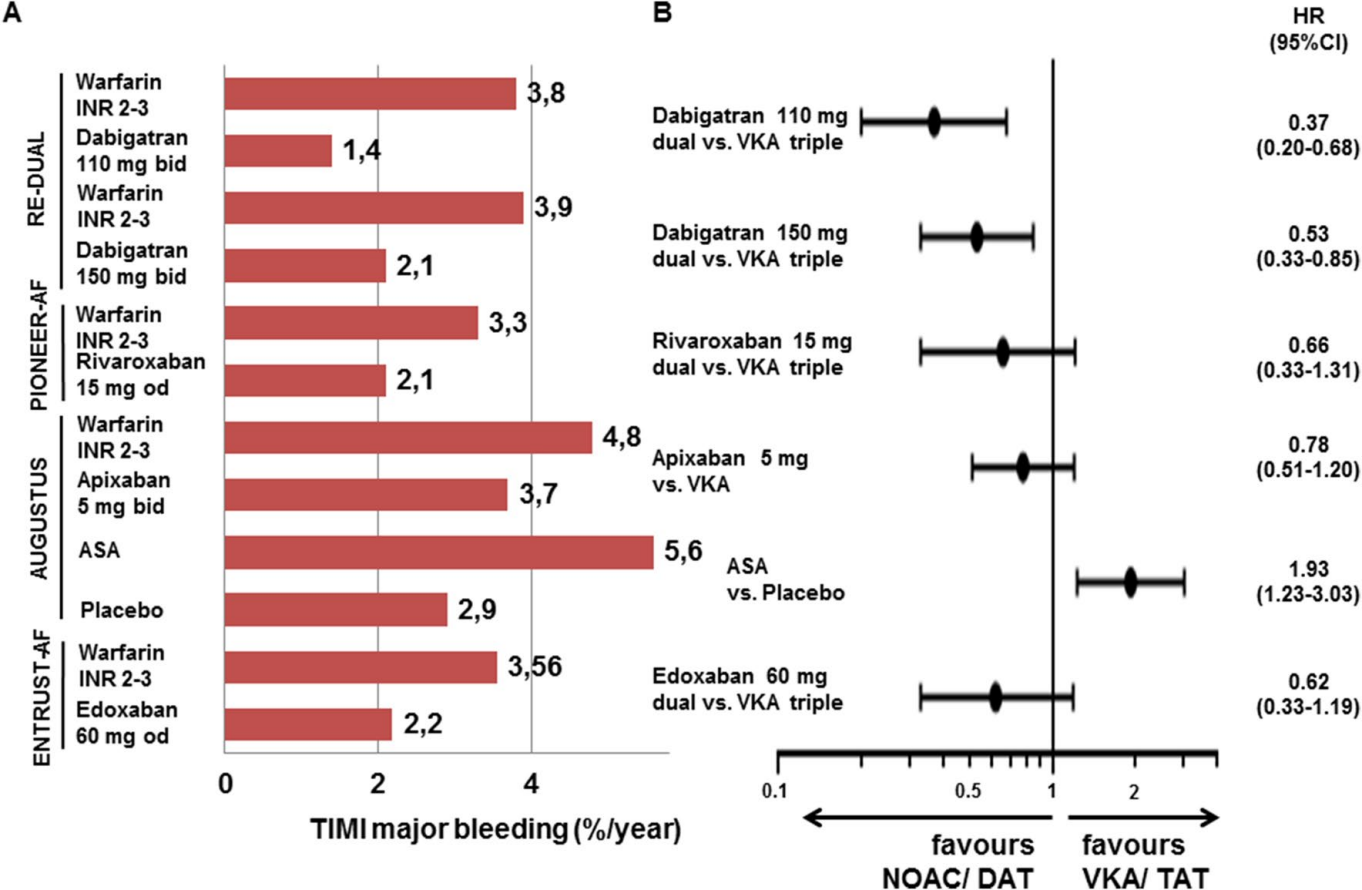
Event rates for TIMI-major bleedings the four NOAC trials using less intense anti-thrombotic strategies following PCI and/or ACS in patients with indication for anticoagulation for stroke prevention in atrial fibrillation. *ASA* acetylsalicylic acid, *DAT* dual anti-thrombotic treatment, *HR* hazard ratio as reported in the trial, *INR* international normaliszed ratio, *NOAC* non-vitamin K oral anticoagulant, *TAT* triple anti-thrombotic treatment, *VKA* vitamin K antagonist

**Fig. 3:**
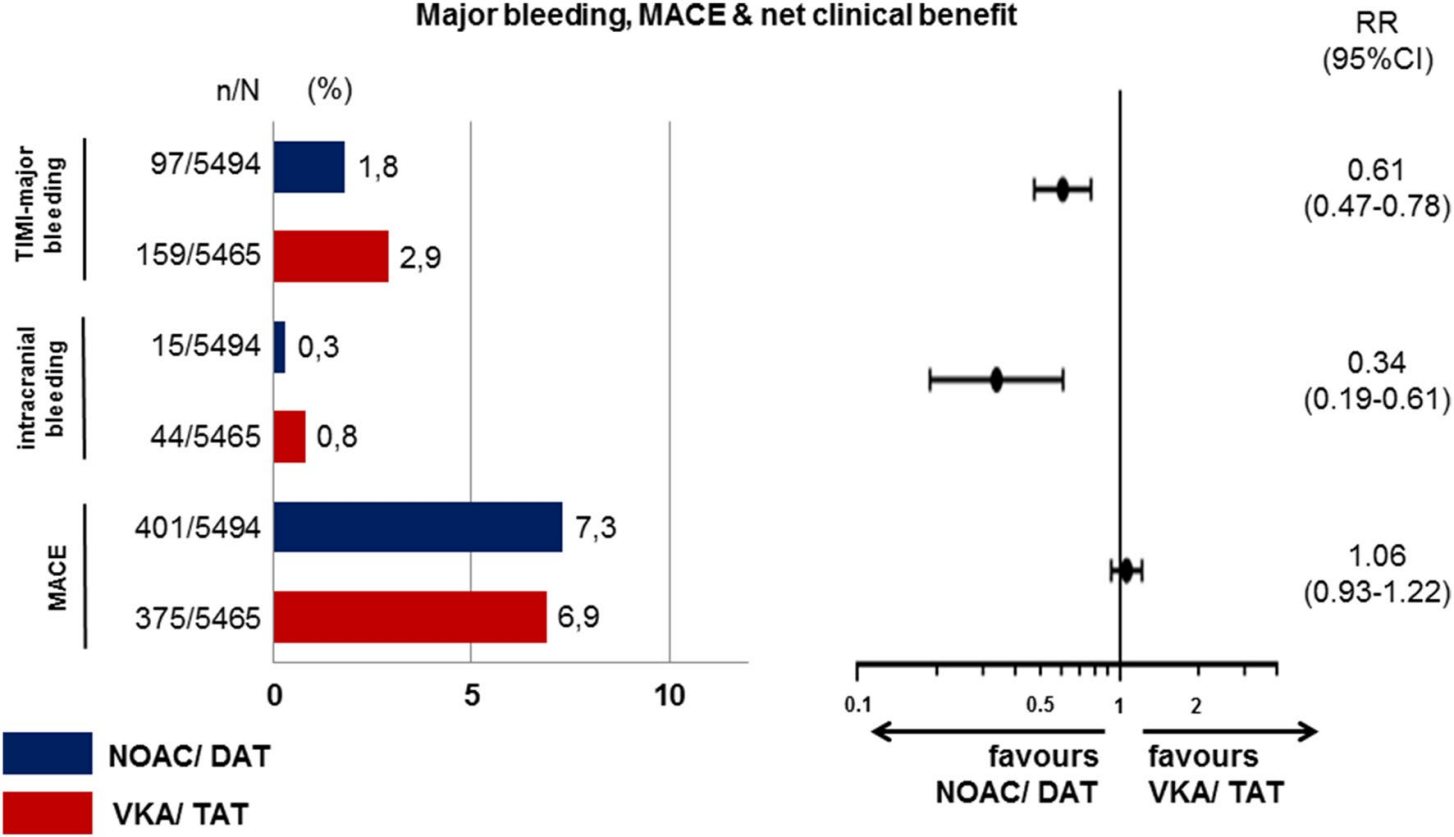
Based on the trials individual events on TIMI-major bleeding, intracranial bleeding as a subgroup of TIMI-major, and MACE were calculated [absolute (*n*) and relative (%) events, patients at risk (*N*), relative risk (RR)] from all NOAC-based strategies compared to VKA-based strategies using less intense anti-thrombotic strategies following PCI and/or ACS in patients with indication for anticoagulation for stroke prevention in atrial fibrillation. *DAT* dual anti-thrombotic treatment, *MACE* major adverse event consisting of cardiovascular death, myocardial infarction (incl. stent thrombosis) and stroke, *NOAC* non-Vitamin K oral anticoagulant, *RR* relative risk, *TAT* triple anti-thrombotic treatment; *VKA* vitamin K antagonist

Due to the rather low number of participants in most of the trials, the primary endpoint was relatively soft considering the composite of major bleeding and clinically-relevant non-major bleedings, which could potentially cause some bias when trying to balance risk and benefit from the primary endpoints. In contrast to previous analyses [60] we focused on primarily reported data on TIMI-major bleedings (Fig. 2). Similarly, efficacy was not even evaluated as a primary endpoint, but in most cases only considered hypothesis generating. Even then, the composite ischemic endpoint was not always restricted to major adverse cardiovascular events (MACE: CV death, non-fatal myocardial infarction and non-fatal stroke), but often included hospitalisations and/or revascularisation. Therefore, we collected data on major bleedings according to TIMI classification (Table 1) with a separate note on intracranial bleedings as well as on MACE (Table 3) from the four trials. The reassuring information on aggregate was that NOAC- compared to VKA-based strategies first reduced the risk for TIMI-major bleeding by 39%, second reduced intracranial bleedings by 66%, and third did not increase MACE rate (Fig. 3). To balance the absolute risk over all trials, we calculated the incremental risk and the incremental benefit over all trials for less intense (NOAC-based dual) compared to more intense (VKA-based triple) anti-thrombotic treatments [61]. While NOAC-based dual strategies were associated overall with an absolute 0.4% incremental risk for MACE, they were also associated with an absolute 1.1% benefit for TIMI-major bleedings compared to VKA-based triple strategies. Therefore, there was an absolute 0.7% risk–benefit [62] in favour of NOAC-based dual strategies based on the higher impact of preventing TIMI-major bleedings compared to MACE, which occurred more often in the population, but did not increase as much during less intense anti-thrombotic treatment. When looking into the randomisation strata of the individual trials, there appears to be a modest risk benefit in most of the analysed strategies (Table 3).

**Table 3:** TIMI-major bleeding, intracranial bleeding and major adverse cardiovascular effects (MACE)

Trial		*n*	TIMI major	ICH	MACE	Incremental risk^a^ ΔMACE	Incremental benefit^b^ ΔTIMI-major	Risk benefit^c^
Re-dual PCI [15]	VKA INR 2–3	981	37 (3.77%)	10 (1.02%)	83 (8.46%)	+ 2.5%	− 2.3%	+ 0.2%
	Dabigatran 110 mg	981	14 (1.43%)	3 (0.31%)	108 (11.01%)			
	VKA INR 2–3	764	30 (3.93%)	8 (1.05%)	60 (7.85%)	± 0.0%	− 1.8%	− 1.8%
	Dabigatran 150 mg	763	16 (2.10%)	1 (0.13%)	60 (7.86%)			
Pioneer AF [16]	VKA INR 2–3	706	20 (2.83%)	4 (0.57%)	36 (5.1%)	+ 0.7%	− 0.9%	− 0.2%
	Rivaroxaban 15 mg	709	14 (1.97%)	2 (0.28%)	41 (5.78%)			
Augustus [17]	VKA INR 2–3	2259	48 (2.12%)	13 (0.58%)	150 (6.64%)	− 0.4%	− 0.5%	− 0.9%
	Apixaban 5 mg	2290	38 (1.66%)	5 (0.22%)	143 (6.24%)			
	ASA	2277	55 (2.42%)	8 (0.35%)	137 (6.02%)	+ 0.8%	− 1.1%	− 0.3%
	Placebo	2279	29 (1.27%)	10 (0.44%)	156 (6.85%)			
Entrust-AF [18]	VKA INR 2–3	755	24 (3.18%)	9 (1.19%)	46 (6.09%)	+ 0.4%	− 1.1%	− 0.7%
	Edoxaban 60 mg	751	15 (2.00%)	4 (0.53%)	49 (6.52%)			
Augustus [52]	Apixaban/placebo	1153	13 (1.13%)	1 (0.09%)	72 (6.24%)	+ 0.5%	− 1.4%	− 0.9%
	VKA/ASS	1154	29 (2.51%)	4 (0.35%)	66 (5.72%)			

^a^Incremental risk indicates the absolute difference in MACE for NOAC-dual vs. VKA-triple anti-thrombotic strategies

^b^Incremental benefit indicates the absolute difference in TIMI-major bleedings for NOAC-dual vs. VKA-triple anti-thrombotic strategies

^c^Risk-benefit is calculated by subtracting the incremental benefit from incremental risk. Positive values indicate higher event rates, negative values indicate lower event rates on NOAC-dual compared to VKA-triple strategies

As the primary data from AUGUSTUS compared VKA to NOAC and independently ASA to placebo, we provide the same calculation for the VKA-triple compared to the apixaban-dual group from data published in a meta-analysis by the AUGUSTUS group [52]. Again, the NOAC-dual arm inherits some risk for more MACE, however, reduction of TIMI-major bleedings was even stronger (Table 3). Recently, a subanalysis from AUGUSTUS indicated that the major prevention of MACE by triple therapy occurred during the first 30 days, whereas triple anti-thrombotic treatment beyond 30 days was related to more significant bleeding events without reducing MACE [56].

## Duration of acetylsalicylic acid and the risk for stent thrombosis

The very early discontinuation of ASA raised the question about an inherent risk of stent thrombosis. PIONEER AF was the only of the four trials that did not have a proper definition and adjudication of stent thrombosis events. Given that limitations, no significant differences were reported between the rivaroxaban dual and the VKA-triple group [0.8% vs 0.7%, HR 1.20 (0.32–4.45)] [16]. RE DUAL-PCI reported definite stent thrombosis to be increased by 86% on the dual dabigatran 110 mg regime [1.5% vs. 0.8%, HR 1.86 (0.79–4.40)] and to be similar on the dual dabigatran 150 mg regime [0.9% vs. 0.9%, HR 0.99 (0.35–2.81)] compared to VKA-triple therapy [15]. AUGUSTUS reported definite or probable stent thrombosis with no relevant difference between apixaban and VKA [1.3%/year vs. 1.6%/year, HR 0.77 (0.38–1.56)] and a marked difference between ASA and placebo [1.0%/year vs. 1.9%/year, HR 0.52 (0.25–1.08)] [17]. Regarding stent thrombosis in AUGUSTUS, at no time had the NOAC been inferior to VKA regarding the occurrence of stent thrombosis and most stent thromboses occurred in the placebo (compared to ASA) group and did so within the first 30 days [63]. ENTRUST AF-PCI reported definite stent thrombosis with some difference between edoxaban dual and VKA-triple regimens [1.16%/year vs. 0.88%/year, HR 1.32 (0.46–3.79)] [18]. Given the low event rates resulting in all trials being underpowered to detect a significant difference, several meta-analyses have been performed [18, 52, 60]. However, the major problem is the reporting of different definitions of stent thrombosis. Some studies do not report probable events, the ones reported from PIONEER AF-PCI as “any” are most likely “definite” ones. If mixing all strategies the question remains whether the lower dose of dabigatran in RE-DUAL PCI should be included in such comparisons. The differing numbers and definitions of stent thrombosis are provided in Table 4. Given the limitations in different definitions of stent thrombosis and mixing those for meta-analyses, there is signal about a hazard ratio of 1.5, which in some analyses is just significant, in others just misses significance. Nevertheless, these aggregate results would suggest that immediate dual anti-thrombotic therapy with ASA only given at the time of procedure might indeed pose some risk regarding stent thrombosis.

**Table 4:** Stent thrombosis in dual vs triple anti-thrombotic treatment trials

Treatment	WOEST		Pioneer AF-PCI			Re-Dual PCI			Augustus				Entrust AF-PCI	
	VKA + P2Y12i	VKA + DAPT	NOAC + P2Y12i	NOAC + DAPT	VKA + DAPT	NOACl + P2Y12i	NOACh + P2Y12i	VKA + DAPT	NOAC + DAPT	NOAC + P2Y12i	VKA + DAPT	VKA + P2Y12i	NOAC + P2Y12i	VKA + DAPT
Efficacy outcome N (ITT)	279	284	694	704	695	981	763	981	1153	1153	1154	1154	751	755
Stent thrombosis definite	1	3	–	–	–	15	7	8	–	–	–	–	8	6
Stent thrombosis definite adn probable	1	5	–	–	–	–	–	–	5	8	6	11	13	10
Stent thrombosis any	4	9	5	6	4	–	–	–	11	21	12	19	–	–

Still the picture remains obscure, as the original AUGUSTUS publication reports two more events than the subsequent publication on stent thrombosis, one each for apixaban and VKA, but both in the placebo group [17, 63]. If one would compare only the full-dose NOAC-based dual to VKA-based triple anti-thrombotic therapies from AUGUSTUS and ENTRUST AF-PCI for definite or probable stent thrombosis, the resulting relative risk ratio is 1.32 (0.69–2.51). When additively including the reported definite stent thrombosis from RE-DUAL PCI in the high-dose group, which is recommended to be used by guidelines now [8], the resulting relative risk ratio for stent thrombosis on full-dose NOAC-based dual compared to VKA-based triple anti-thrombotic therapies is 1.22 (0.70–2.11). The overall rate of stent thrombosis in all trials is reassuringly low. The number needed to treat to prevent one stent thrombosis by adding ASA in AUGUSTUS was 250. The number needed to harm to induce one major bleeding by adding ASA in AUGUSTUS was 55 using the ISTH definition and 91 using the TIMI definition [17]. Therefore, even eventually significant increases in relative risk for stent thrombosis by NOAC-based dual compared to VKA-based triple anti-thrombotic therapies might not translate into clinical net harm as more major bleedings are prevented at the same time by dual anti-thrombotic regimens. This thought is supported by the clinical net-benefit analyses.

In summary, the accumulated data suggest that ASA beyond 30 days is not required in patients on OAC plus a P2Y_12_ inhibitor following PCI. For the first month, however, severe bleeding and ischemic events have to be weighed against each other and the treatment regimen has to be adopted on an individual basis including patient-specific ischemic and bleeding risk.

## The influence of more potent P2Y_12_ inhibition

Current guidelines state that “the use of ticagrelor or prasugrel is not recommended as part of triple anti-thrombotic therapy with aspirin and an OAC” [8]. However, there is no clear recommendation regarding those P2Y_12_ inhibitors, which are more potent than clopidogrel, in the context of dual anti-thrombotic therapy, e.g. when ASA is dropped very early. Their use in the contemporary OAC PCI trials was low (Fig. 1) and most probably the patients treated with them might have had different baseline characteristics (e.g. younger, lower bleeding risk) compared to patients being treated with clopidogrel. Therefore, one has to be very cautious when interpreting reported bleeding rates, which were sometimes higher, sometimes lower on more potent P2Y_12_ inhibitors added to OAC (Suppl. Table 1). One major consistent finding through all four trials, however, was that the bleeding on the more potent P2Y_12_ blockers was always lower in the NOAC/dual compared to the VKA/triple strategy,

## Conclusion

In general the available data from all studies suggest that bleeding can be lowered significantly by reducing the intensity of anti-thrombotic treatment. In PCI patients with AF as an indication for OAC, NOACs in the same dose as approved for stroke prevention have shown the most reliable protection regarding stroke prevention and coronary ischaemia. NOAC-based strategies inherit a lower risk for major bleedings than VKA-based regimes and should, therefore, be preferred. Beyond 30 days, there are no conclusive data supporting triple therapy with additive ASA. For the first month, there are concerns regarding potential stent thrombosis, even so the rates appear to be low. Dedicated analyses from AUGUSTUS suggest that individual balancing of ischaemic vs bleeding risk is required to tailor the optimal anti-thrombotic regimen for AF-PCI patients requiring OAC for stroke prevention. Regarding elderly patients with higher stroke risk, strategies with deliberate dose reductions should be avoided as they pose a higher risk for ischemic/ embolic events.

## Electronic supplementary material

Below is the link to the electronic supplementary material.

Supplementary file1 (DOCX 22 kb)

## References

[1] Capodanno D, Huber K, Mehran R, Lip GYH, Faxon DP, Granger CB (2019). Management of Antithrombotic Therapy in Atrial Fibrillation Patients Undergoing PCI: JACC State-of-the-Art Review. J Am Coll Cardiol.

[2] Granger CB, Armaganijan LV (2012). Newer oral anticoagulants should be used as first-line agents to prevent thromboembolism in patients with atrial fibrillation and risk factors for stroke or thromboembolism. Circulation.

[3] Hylek EM, Go AS, Chang Y, Jensvold NG, Henault LE, Selby JV (2003). Effect of intensity of oral anticoagulation on stroke severity and mortality in atrial fibrillation. N Engl J Med.

[4] Leon MB, Baim DS, Popma JJ, Gordon PC, Cutlip DE, Ho KK et al (1998) A clinical trial comparing three antithrombotic-drug regimens after coronary-artery stenting. Stent Anticoagulation Restenosis Study Investigators. N Engl J Med 339(23):1665–1671. 10.1056/NEJM199812033392303 10.1056/NEJM199812033392303 9834303

[5] Schomig A, Neumann FJ, Kastrati A, Schuhlen H, Blasini R, Hadamitzky M (1996). A randomized comparison of antiplatelet and anticoagulant therapy after the placement of coronary-artery stents. N Engl J Med.

[6] Investigators AWGotA, Connolly S, Pogue J, Hart R, Pfeffer M, Hohnloser S et al (2006) Clopidogrel plus aspirin versus oral anticoagulation for atrial fibrillation in the Atrial fibrillation Clopidogrel Trial with Irbesartan for prevention of Vascular Events (ACTIVE W): a randomised controlled trial. Lancet 367(9526):1903–1912. 10.1016/S0140-6736(06)68845-4 10.1016/S0140-6736(06)68845-4 16765759

[7] January CT, Wann LS, Calkins H, Chen LY, Cigarroa JE, Cleveland JC (2019). 2019 AHA/ACC/HRS Focused Update of the 2014 AHA/ACC/HRS guideline for the management of patients with atrial fibrillation: a report of the American College of Cardiology/American Heart Association Task Force on Clinical Practice Guidelines and the Heart Rhythm Society in Collaboration With the Society of Thoracic Surgeons. Circulation.

[8] Neumann FJ, Sousa-Uva M, Ahlsson A, Alfonso F, Banning AP, Benedetto U (2019). 2018 ESC/EACTS Guidelines on myocardial revascularization. Eur Heart J.

[9] Kirchhof P, Benussi S, Kotecha D, Ahlsson A, Atar D, Casadei B (2016). 2016 ESC Guidelines for the management of atrial fibrillation developed in collaboration with EACTS. Eur Heart J.

[10] Hansen ML, Sorensen R, Clausen MT, Fog-Petersen ML, Raunso J, Gadsboll N (2010). Risk of bleeding with single, dual, or triple therapy with warfarin, aspirin, and clopidogrel in patients with atrial fibrillation. Arch Intern Med.

[11] Mega JL, Braunwald E, Mohanavelu S, Burton P, Poulter R, Misselwitz F (2009). Rivaroxaban versus placebo in patients with acute coronary syndromes (ATLAS ACS-TIMI 46): a randomised, double-blind, phase II trial. Lancet.

[12] Alexander JH, Lopes RD, James S, Kilaru R, He Y, Mohan P (2011). Apixaban with antiplatelet therapy after acute coronary syndrome. N Engl J Med.

[13] Morrow DA, Braunwald E, Bonaca MP, Ameriso SF, Dalby AJ, Fish MP (2012). Vorapaxar in the secondary prevention of atherothrombotic events. N Engl J Med.

[14] Oldgren J, Budaj A, Granger CB, Khder Y, Roberts J, Siegbahn A et al (2011) Dabigatran vs. placebo in patients with acute coronary syndromes on dual antiplatelet therapy: a randomized, double-blind, phase II trial. Eur Heart J 32(22):2781–2789. 10.1093/eurheartj/ehr113 10.1093/eurheartj/ehr113 21551462

[15] Cannon CP, Bhatt DL, Oldgren J, Lip GYH, Ellis SG, Kimura T (2017). Dual antithrombotic therapy with dabigatran after PCI in atrial fibrillation. N Engl J Med.

[16] Gibson CM, Mehran R, Bode C, Halperin J, Verheugt FW, Wildgoose P (2016). Prevention of bleeding in patients with atrial fibrillation undergoing PCI. N Engl J Med.

[17] Lopes RD, Heizer G, Aronson R, Vora AN, Massaro T, Mehran R (2019). Antithrombotic therapy after acute coronary syndrome or PCI in atrial fibrillation. N Engl J Med.

[18] Vranckx P, Valgimigli M, Eckardt L, Tijssen J, Lewalter T, Gargiulo G (2019). Edoxaban-based versus vitamin K antagonist-based antithrombotic regimen after successful coronary stenting in patients with atrial fibrillation (ENTRUST-AF PCI): a randomised, open-label, phase 3b trial. Lancet.

[19] Valgimigli M, Bueno H, Byrne RA, Collet J-P, Costa F, Jeppsson A (2018). 2017 ESC focused update on dual antiplatelet therapy in coronary artery disease developed in collaboration with EACTS. Eur Heart J.

[20] Roffi M, Patrono C, Collet JP, Mueller C, Valgimigli M, Andreotti F (2016). 2015 ESC Guidelines for the management of acute coronary syndromes in patients presenting without persistent ST-segment elevation: Task Force for the Management of Acute Coronary Syndromes in Patients Presenting without Persistent ST-Segment Elevation of the European Society of Cardiology (ESC). Eur Heart J.

[21] Ibanez B, James S, Agewall S, Antunes MJ, Bucciarelli-Ducci C, Bueno H (2018). 2017 ESC guidelines for the management of acute myocardial infarction in patients presenting with ST-segment elevation. Eur Heart J.

[22] Valgimigli M, Patialiakas A, Thury A, McFadden E, Colangelo S, Campo G (2015). Zotarolimus-eluting versus bare-metal stents in uncertain drug-eluting stent candidates. J Am Coll Cardiol.

[23] Haeusler KG, Groschel K, Kohrmann M, Anker SD, Brachmann J, Bohm M (2018). Expert opinion paper on atrial fibrillation detection after ischemic stroke. Clin Res Cardiol.

[24] Steffel J, Verhamme P, Potpara TS, Albaladejo P, Antz M, Desteghe L (2018). The 2018 European Heart Rhythm Association Practical Guide on the use of non-vitamin K antagonist oral anticoagulants in patients with atrial fibrillation. Eur Heart J.

[25] Sharma M, Cornelius VR, Patel JP, Davies JG, Molokhia M (2015). Efficacy and harms of direct oral anticoagulants in the elderly for stroke prevention in atrial fibrillation and secondary prevention of venous thromboembolism: systematic review and meta-analysis. Circulation.

[26] Koziel M, Al-Saady N, Hjortshoj SP, Goudev A, Huber K, Cohen A (2020). Edoxaban versus warfarin in vitamin K antagonist experienced and naive patients from the edoxaban versus warfarin in subjects undergoing cardioversion of atrial fibrillation (ENSURE-AF) randomised trial. Clin Res Cardiol.

[27] Goette A, Merino JL, Ezekowitz MD, Zamoryakhin D, Melino M, Jin J (2016). Edoxaban versus enoxaparin-warfarin in patients undergoing cardioversion of atrial fibrillation (ENSURE-AF): a randomised, open-label, phase 3b trial. Lancet.

[28] Ezekowitz MD, Pollack CV, Halperin JL, England RD, VanPelt NS, Spahr J (2018). Apixaban compared to heparin/vitamin K antagonist in patients with atrial fibrillation scheduled for cardioversion: the EMANATE trial. Eur Heart J.

[29] Kottmaier M, Baur A, Lund S, Bourier F, Reents T, Semmler V (2020). Atrial fibrillation ablation in adults with congenital heart disease on uninterrupted oral anticoagulation is safe and efficient. Clin Res Cardiol.

[30] Cappato R, Marchlinski FE, Hohnloser SH, Naccarelli GV, Xiang J, Wilber DJ et al (2015) Uninterrupted rivaroxaban vs. uninterrupted vitamin K antagonists for catheter ablation in non-valvular atrial fibrillation. Eur Heart J 36(28):1805–1811. 10.1093/eurheartj/ehv177 10.1093/eurheartj/ehv177 PMC4508487 25975659

[31] Kirchhof P, Haeusler KG, Blank B, De Bono J, Callans D, Elvan A (2018). Apixaban in patients at risk of stroke undergoing atrial fibrillation ablation. Eur Heart J.

[32] Hohnloser SH, Camm J, Cappato R, Diener HC, Heidbuchel H, Mont L et al (2019) Uninterrupted edoxaban vs. vitamin K antagonists for ablation of atrial fibrillation: the ELIMINATE-AF trial. Eur Heart J 40(36):3013–3021. 10.1093/eurheartj/ehz190 10.1093/eurheartj/ehz190 PMC6754569 30976787

[33] Geis NA, Kiriakou C, Chorianopoulos E, Uhlmann L, Katus HA, Bekeredjian R (2018). NOAC monotherapy in patients with concomitant indications for oral anticoagulation undergoing transcatheter aortic valve implantation. Clin Res Cardiol.

[34] Nelles D, Lambers M, Schafigh M, Morais P, Schueler R, Vij V (2020). Clinical outcomes and thrombus resolution in patients with solid left atrial appendage thrombi: results of a single-center real-world registry. Clin Res Cardiol.

[35] Lip GY, Hammerstingl C, Marin F, Cappato R, Meng IL, Kirsch B (2016). Left atrial thrombus resolution in atrial fibrillation or flutter: Results of a prospective study with rivaroxaban (X-TRA) and a retrospective observational registry providing baseline data (CLOT-AF). Am Heart J.

[36] Hohnloser SH, Basic E, Nabauer M (2019). Uptake in antithrombotic treatment and its association with stroke incidence in atrial fibrillation: insights from a large German claims database. Clin Res Cardiol.

[37] Eggebrecht L, Prochaska JH, Trobs SO, Schwuchow-Thonke S, Gobel S, Diestelmeier S (2019). Direct oral anticoagulants and vitamin K antagonists are linked to differential profiles of cardiac function and lipid metabolism. Clin Res Cardiol.

[38] Connolly SJ, Eikelboom J, Joyner C, Diener HC, Hart R, Golitsyn S (2011). Apixaban in patients with atrial fibrillation. N Engl J Med.

[39] Ng KH, Shestakovska O, Connolly SJ, Eikelboom JW, Avezum A, Diaz R (2016). Efficacy and safety of apixaban compared with aspirin in the elderly: a subgroup analysis from the AVERROES trial. Age Ageing.

[40] Goette A, Merino JL, De Caterina R, Huber K, Heidbuchel H, Jin J (2020). Effect of concomitant antiplatelet agents on clinical outcomes in the edoxaban vs warfarin in subjects undergoing cardioversion of atrial fibrillation (ENSURE-AF) randomized trial. Clin Res Cardiol.

[41] Fischbach W, Malfertheiner P, Lynen Jansen P, Bolten W, Bornschein J, Buderus S (2016). S2k-guideline *Helicobacter pylori* and gastroduodenal ulcer disease. Z Gastroenterol.

[42] Abraham NS, Hlatky MA, Antman EM, Bhatt DL, Bjorkman DJ, Clark CB (2010). ACCF/ACG/AHA 2010 Expert Consensus Document on the concomitant use of proton pump inhibitors and thienopyridines: a focused update of the ACCF/ACG/AHA 2008 expert consensus document on reducing the gastrointestinal risks of antiplatelet therapy and NSAID use: a report of the American College of Cardiology Foundation Task Force on Expert Consensus Documents. Circulation.

[43] Sarafoff N, Martischnig A, Wealer J, Mayer K, Mehilli J, Sibbing D (2013). Triple therapy with aspirin, prasugrel, and vitamin K antagonists in patients with drug-eluting stent implantation and an indication for oral anticoagulation. J Am Coll Cardiol.

[44] Ohman EM, Roe MT, Steg PG, James SK, Povsic TJ, White J (2017). Clinically significant bleeding with low-dose rivaroxaban versus aspirin, in addition to P2Y12 inhibition, in acute coronary syndromes (GEMINI-ACS-1): a double-blind, multicentre, randomised trial. Lancet.

[45] Connolly SJ, Ezekowitz MD, Yusuf S, Eikelboom J, Oldgren J, Parekh A (2009). Dabigatran versus warfarin in patients with atrial fibrillation. N Engl J Med.

[46] Artang R, Rome E, Nielsen JD, Vidaillet HJ (2013). Meta-analysis of randomized controlled trials on risk of myocardial infarction from the use of oral direct thrombin inhibitors. Am J Cardiol.

[47] Petzold T, Thienel M, Konrad I, Schubert I, Regenauer R, Hoppe B et al (2016) Oral thrombin inhibitor aggravates platelet adhesion and aggregation during arterial thrombosis. Sci Transl Med 8(367):367ra168. 10.1126/scitranslmed.aad6712 10.1126/scitranslmed.aad6712 27903864

[48] Mega JL, Braunwald E, Wiviott SD, Bassand JP, Bhatt DL, Bode C (2012). Rivaroxaban in patients with a recent acute coronary syndrome. N Engl J Med.

[49] Dewilde WJ, Oirbans T, Verheugt FW, Kelder JC, De Smet BJ, Herrman JP (2013). Use of clopidogrel with or without aspirin in patients taking oral anticoagulant therapy and undergoing percutaneous coronary intervention: an open-label, randomised, controlled trial. Lancet.

[50] Duerschmied D, Brachmann J, Darius H, Frey N, Katus HA, Rottbauer W (2018). Antithrombotic therapy in patients with non-valvular atrial fibrillation undergoing percutaneous coronary intervention: should we change our practice after the PIONEER AF-PCI and RE-DUAL PCI trials?. Clin Res Cardiol.

[51] Wernly B, Lichtenauer M, Erlinge D, Jung C (2020). Antithrombotic therapy in atrial fibrillation: stop triple therapy and start optimizing dual therapy?. Clin Res Cardiol.

[52] Lopes RD, Hong H, Harskamp RE, Bhatt DL, Mehran R, Cannon CP (2019). Safety and efficacy of antithrombotic strategies in patients with atrial fibrillation undergoing percutaneous coronary intervention: a network meta-analysis of randomized controlled trials. JAMA Cardiol.

[53] Chesebro JH, Knatterud G, Roberts R, Borer J, Cohen LS, Dalen J (1987). Thrombolysis in myocardial infarction (TIMI) Trial, Phase I: A comparison between intravenous tissue plasminogen activator and intravenous streptokinase. Clinical findings through hospital discharge. Circulation.

[54] Kaatz S, Ahmad D, Spyropoulos AC, Schulman S, Subcommittee on Control of A (2015) Definition of clinically relevant non-major bleeding in studies of anticoagulants in atrial fibrillation and venous thromboembolic disease in non-surgical patients: communication from the SSC of the ISTH. J Thromb Haemost 13(11):2119–2126 10.1111/jth.13140 26764429

[55] Granger CB, Alexander JH, McMurray JJ, Lopes RD, Hylek EM, Hanna M (2011). Apixaban versus warfarin in patients with atrial fibrillation. N Engl J Med.

[56] Alexander JH, Wojdyla D, Vora AN, Thomas L, Granger CB, Goodman SG (2020). The risk/benefit tradeoff of antithrombotic therapy in patients with atrial fibrillation early and late after an acute coronary syndrome or percutaneous coronary intervention: insights from AUGUSTUS. Circulation.

[57] Vranckx P, Lewalter T, Valgimigli M, Tijssen JG, Reimitz PE, Eckardt L (2018). Evaluation of the safety and efficacy of an edoxaban-based antithrombotic regimen in patients with atrial fibrillation following successful percutaneous coronary intervention (PCI) with stent placement: Rationale and design of the ENTRUST-AF PCI trial. Am Heart J.

[58] Jobski K, Hoffmann F, Herget-Rosenthal S, Dorks M (2020). Drug interactions with oral anticoagulants in German nursing home residents: comparison between vitamin K antagonists and non-vitamin K antagonist oral anticoagulants based on two nested case-control studies. Clin Res Cardiol.

[59] Schäfer A, Flierl U, Berliner D, Bauersachs J (2020). Anticoagulants for stroke prevention in atrial fibrillation in elderly patients. Cardiovasc Drug Ther.

[60] Gargiulo G, Goette A, Tijssen J, Eckardt L, Lewalter T, Vranckx P et al (2019) Safety and efficacy outcomes of double vs. triple antithrombotic therapy in patients with atrial fibrillation following percutaneous coronary intervention: a systematic review and meta-analysis of non-vitamin K antagonist oral anticoagulant-based randomized clinical trials. Eur Heart J 40(46):3757–3767. 10.1093/eurheartj/ehz732 10.1093/eurheartj/ehz732 31651946

[61] Lynd LD, O'Brien BJ (2004). Advances in risk-benefit evaluation using probabilistic simulation methods: an application to the prophylaxis of deep vein thrombosis. J Clin Epidemiol.

[62] Singer DE, Chang Y, Fang MC, Borowsky LH, Pomernacki NK, Udaltsova N (2009). The net clinical benefit of warfarin anticoagulation in atrial fibrillation. Ann Intern Med.

[63] Lopes RD, Leonardi S, Wojdyla DM, Vora AN, Thomas L, Storey RF (2020). Stent thrombosis in patients with atrial fibrillation undergoing coronary stenting in the AUGUSTUS Trial. Circulation.

[64] Mehran R, Rao SV, Bhatt DL, Gibson CM, Caixeta A, Eikelboom J (2011). Standardized bleeding definitions for cardiovascular clinical trials: a consensus report from the Bleeding Academic Research Consortium. Circulation.

[65] Investigators G (1993) An international randomized trial comparing four thrombolytic strategies for acute myocardial infarction. N Engl J Med 329(10):673–682. 10.1056/NEJM199309023291001 10.1056/NEJM199309023291001 8204123

[66] Schulman S, Kearon C, Subcommittee on Control of Anticoagulation of the S, Standardization Committee of the International Society on T, Haemostasis (2005) Definition of major bleeding in clinical investigations of antihemostatic medicinal products in non-surgical patients. J Thromb Haemost 3(4):692–694. 10.1111/j.1538-7836.2005.01204.x 10.1111/j.1538-7836.2005.01204.x 15842354

[67] Wallentin L, Becker RC, Budaj A, Cannon CP, Emanuelsson H, Held C (2009). Ticagrelor versus clopidogrel in patients with acute coronary syndromes. N Engl J Med.

