# Supplementary material for: Anti-thrombotic strategies in patients with atrial fibrillation undergoing PCI
Source: Clin Res Cardiol. 2020 Jul 21;110(6):759–74. doi: 10.1007/s00392-020-01708-8 (PMC8166702; doi:10.1007/s00392-020-01708-8)
Supplement: Supplementary file 1 — Supplementary file1 (DOCX 22 kb) [file 392_2020_1708_MOESM1_ESM.docx]

**Suppl. Table 1: Primary bleeding endpoints on more potent P2Y12 inhibitors (Prasugrel/Ticagrelor) compared to clopidogrel**

| **trial** |  | **Clopidogrel** | **Prasugrel/Ticagrelor** |
| --- | --- | --- | --- |
| **RE-DUAL PCI[15]** | **VKA**  **INR 2-3** | **230/890**  **(26%)** | **34/91**  **(37%)** |
|  | **Dabigatran**  **110 mg** | **123/849**  **(15%)** | **28/132**  **(21%)** |
|  | **VKA**  **INR 2-3** | **171/691**  **(25%)** | **25/73**  **(34%)** |
|  | **Dabigatran**  **150 mg** | **130/659**  **(20%)** | **24/104**  **(23%)** |
| **PIONEER-AF[16]** | **VKA**  **INR 2-3** | **157/671**  **(26%)** | **10/36**  **(28%)** |
|  | **Rivaroxaban**  **15 mg** | **99/648**  **(16%)** | **10/48**  **(21%)** |
| **AUGUSTUS[17]**  ***** | **VKA**  **INR 2-3** | **499/4106**  **(12%)** | **61/328**  **(19%)** |
|  | **Apixaban**  **5 mg** |  |  |
| **ENTRUST-AF[18]** | **VKA**  **INR 2-3** | **141/695**  **(21%)** | **11/60**  **(22%)** |
|  | **Edoxaban**  **60 mg** | **121/696**  **(21%)** | **7/54**  **(14%)** |

*the reported hazard ratio for the primary bleeding endpoint for apixaban vs VKA was 0.67 for clopidogrel, 0.75 for prasugrel, and 0.84 for ticagrelor
